# Capacity development for health research in Africa: experiences managing the African Doctoral Dissertation Research Fellowship Program

**DOI:** 10.1186/1478-4505-8-21

**Published:** 2010-06-29

**Authors:** Caroline W Kabiru, Chimaraoke O Izugbara, Susan W Wambugu, Alex C Ezeh

**Affiliations:** 1 African Population and Health Research Center, 2nd Floor Shelter Afrique Centre, P.O. Box 10787-00100, Nairobi, Kenya

## Abstract

Africa's progress depends on her capacity to generate, adapt, and use scientific knowledge to meet regional health and development needs. Yet, Africa's higher education institutions that are mandated to foster this capacity lack adequate resources to generate and apply knowledge, raising the need for innovative approaches to enhance research capacity. In this paper, we describe a newly-developed program to support PhD research in health and population sciences at African universities, the African Doctoral Dissertation Research Fellowship (ADDRF) Program. We also share our experiences implementing the program. As health research capacity-strengthening in Africa continues to attract attention and as the need for such programs to be African-led is emphasized, our experiences in developing and implementing the ADDRF offer invaluable lessons to other institutions undertaking similar initiatives.

## Introduction

Several challenges face university education in sub-Saharan Africa. Unprecedented growth in student enrolment, rising from 337,000 in 1980 to an estimated 4,000,000 in 2004 and the expansion of training programs, especially at the undergraduate level, have occurred at a time when per capita funding for universities is being reduced [1]. Many universities in the region presently operate with overburdened and underpaid faculty who often resort to consultancies to maintain a basic living standard [2]. The increasing exodus of human capital from academic and research sectors in Africa adds to the continent's decreasing contribution to global scientific output as well as the widening gap in science and technology between Africa and the rest of the world [3]. While the above problems affect university programs in Africa across the board, their impact on graduate studies, and particularly, doctoral training has been disproportionate.

Students in graduate programs on the continent lack role models and mentors, strong academic and research networks, opportunities to participate in international conferences, and exposure to strong research environments. Further, many graduate students on the continent lack funding for their studies and are forced to work part-time, consequently taking many years to graduate. The declining quality of products from graduate programs in Africa is already telling as evidenced, among other things, by frequent complaints by employers regarding the competence and ability of the graduates [4]. To a very large extent, however, Africa's progress will depend on her capacity to nurture researchers and thinkers who are able 'to understand, interpret, select, adapt, use, transmit, diffuse, produce, and commercialize scientific knowledge in ways appropriate to its culture, aspirations, and level of development' (p.iv) [5]. Underscoring the importance of quality doctoral training, Szanton and Manyika [6] contend that 'it is essential for every country to have a large and growing cadre of highly skilled professionals; thinkers, actors, writers, teachers, male and female, in a wide range of fields who are capable of producing critical analyses, local and national policies, and programs to deal with the internal and external social and cultural issues facing their nation' (p.41).

The great need facing graduate-level training in Africa creates an enormous opportunity for innovative interventions. Indeed, several international agencies and funding bodies now provide a wide range of fellowships to support African PhD students. For example, the International Development Research Centre (IDRC) in Canada runs the Southern Junior Researchers Awards program which primarily supports PhD studies in developing countries, with a focus on sub-Saharan Africa [7]. The UK Department for International Development (DfID) supports awards for developing countries under the Commonwealth Scholarships and Fellowships Awards. This funding scheme supports post-graduate studies for citizens from one Commonwealth country who are pursuing post-graduate education in another Commonwealth country [8]. The Wellcome Trust provides Masters Fellowships in Public Health and Tropical Medicine, which though targeted towards Masters students, can be applied for PhD studies for exceptional candidates with the Masters Fellowships [9]. Finally, the Ford Foundation supports the International Fellowship Program (IFP), which provides fellowships to support post-graduate studies in the Foundation's grant-making areas for citizens of IFP countries or territories (Brazil, Chile, China, Egypt, Ghana, Guatemala, India, Indonesia, Kenya, Mexico, Mozambique, Nigeria, Palestinian Territories, Peru, Philippines, Russia, Senegal, South Africa, Tanzania, Thailand, Uganda, and Vietnam) [10]. Other efforts to strengthen health research capacity in Africa include the Wellcome Trust's African Institutions Initiative - a £30 M initiative launched in 2009 and supporting seven different consortia and involving 50 research institutions and universities in 18 African and six northern countries [11]; the Wellcome Trust, DfID and IDRC funded Health Research Capacity Strengthening (HRCS) initiative in Kenya and Malawi [12]; and the EU FP-7-AFRICA-2010 call with specific focus on building sustainable capacity for research for health in Africa [13].

In this paper, we provide an overview of one response to the challenges facing graduate-level training in Africa: the African Doctoral Dissertation Research Fellowship (ADDRF) Program, a program funded under IDRC's Southern Junior Researchers Awards program. The aims of the program are to facilitate rigorous research addressing health issues in Africa, enhance the quality of doctoral dissertation research, and equip doctoral students with essential research skills. Initiated in 2008 with funding from the IDRC and the Ford Foundation, the ADDRF is now in its third year of operation. As capacity-strengthening for health research continues to receive attention in Africa, our experiences as implementers of the ADDRF offer funders and research capacity-strengthening institutions something to learn from.

## Discussion

### Program Description

The African Doctoral Dissertation Research Fellowships are awarded to advanced doctoral students who are citizens or permanent residents of a sub-Saharan African country and are within two years of completing their doctoral thesis at an African university. The fellowships target students whose research shows great promise of making significant contributions to governance, equity, sexuality, health or population-related issues in the region. In particular, the fellowship program aims to bridge the knowledge gap in health systems research in sub-Saharan Africa. The program's general objectives are to facilitate more rigorous engagement of doctoral students in research; to provide the Fellows an opportunity for timely completion of their doctoral training; and to strengthen doctoral students' research skills.

Each fellowship (with a maximum award of US $15,000) includes a modest monthly stipend, funds to support data collection and analyses, as well as support to attend a regional or international conference. Anecdotal reports indicate that the dissertation review process in many African universities is long, laborious, and often delays graduation by years. Thus, where needed, candidates' home departments are also provided with modest facilitation grants to enable them provide effective and timely supervision to the grant recipients and to facilitate internal and external reviews of students' completed dissertations. As part of the award, all grant recipients participate in two training workshops. The program has managed to award more fellowships than budgeted because students are not always funded at the full amount. Consequently, the estimated total cost per student taking into account the fellowship award, institutional support, workshop and other administrative costs is US $25,000.

The training workshops are intended to introduce students to research methods and ethics, literature retrieval, reference management, scientific writing, proposal development, and communication of research. The workshops also serve as a networking opportunity for cohorts of Fellows which is hoped to strengthen future collaborations across national boundaries. The content for these workshops is largely informed by areas of training needs highlighted by the Fellows.

To qualify for the award, applicants must have completed all pre-dissertation requirements and show evidence of being able to graduate within 24 months of the start of the fellowship. As part of the application process, applicants submit a scientific proposal detailing the research question(s), policy-relevance, study design, and a statement of future research interests. They also provide a detailed time-frame for completing their dissertation which is endorsed by the head of department or the chair of the dissertation committee. Applicants also provide evidence that their research topic has been approved by their doctoral committee, and that the study has received ethical clearance. The fellowship is awarded only once to a doctoral student.

Fellowship funds are disbursed in three installments. However, to reduce administrative costs and ensure greater flexibility for successful candidates to apply the funds in ways that enhance their work, a substantial proportion of the grant amount is paid at the outset with the rest tied to meeting specific milestones. The final payout is expected to be released upon approval of the dissertation by the department.

### Management of the program

The ADDRF program is managed by the African Population and Health Research Centre (APHRC) based in Nairobi, Kenya. The ADDRF team works closely with other departments and staff at the Center. These include the finance department (for administration of the awards), logistics staff (for organization of the workshops and trainings), and researchers at the Center who assist with the review process and serve as mentors to Fellows or applicants whose proposals are not funded, but show great promise.

### Selection process

For the first two rounds of application reviews, applications were evaluated on the following criteria (maximum score in parentheses): candidate's scientific background and potential for development of a strong research career (20%); scientific merit of the proposed research project including originality of research question; clear study design including a description of sampling methods and considerations, tools for data collection, data management and quality assurance; demonstrated knowledge of relevant and current literature; detailed analysis plan, etc. (40%); research environment including departmental commitment to facilitate timely completion of the dissertation (20%); well-elaborated statement on the policy relevance of the research (10%); and budget summary and justification, including clear plan to complete the dissertation within 24 months (10%). For the third round, applications were evaluated on three criteria: the candidate's scientific background and potential for development of a strong research career (20%); the scientific merit of the proposed research project (60%); and the research training environment (20%).

Narrowing the large number of proposals received to the small proportion that gets funded requires a rigorous review process. To do this, we engage reviewers who are reputable academics with extensive experience in doctoral training and/or research on governance, equity, health and population-related issues in Africa. Before applications are sent to reviewers, a preliminary assessment is undertaken to ensure completeness and adherence both to the application guidelines and the spirit of the fellowship.

Each proposal is evaluated by three reviewers, at least one of whom must serve on the selection committee. One of the reviewers must also have substantive knowledge of the applicant's disciplinary background. Each reviewer scores a given proposal and makes written recommendations as to whether the proposal should be funded, discussed, or rejected.

The selection committee, comprising some of the reviewers, then meets face-to-face to review the scores and recommendations and makes a final agreement on which proposals should be funded. Generally, where all three reviewers are in agreement to reject a proposal, the proposal is rejected with little discussion. Depending on the number of proposals in which the committee is in agreement to fund, proposals are then ranked. While emphasizing the quality of the proposed research project and suitability of the candidate, the selection committee takes into consideration the country of origin and gender of the applicants to ensure balanced regional (Anglophone and Francophone) and gender representation of grantees. However, grants are awarded nonetheless to other qualified applicants if no suitable applicant with the expected regional or gender attributes is found.

In its first year (2008), the fellowship program supported 20 (12 females) doctoral students from eight African countries studying health-issues or matters related to sexuality. Two of these fellowships were supported by the Ford Foundation. In its second year, an addition 25 students (three of whom are supported by the Ford Foundation) representing 13 sub-Saharan African countries and 16 African universities have received support. In 2010, 19 students (three of whom are supported by the Ford Foundation) were awarded fellowships.

### Key lessons

Administratively, our initial experience brought to light the importance of establishing a system to facilitate the review process. In the first instance, we simply requested students to submit key documents (research proposal, proof of residency, budget, and evidence of ethical clearance to conduct their research). As a result, applications were received in different formats and did not always include key supporting documents, creating substantial challenges for reviewers. For the second call, we required that all applications be submitted using a pre-defined template to ensure uniformity and completeness. Although this alleviated some of the challenges, the template needed further refinement to incorporate information on other sources of funding available to the applicant, at what stage the study is, institutional capacity, and budget justification. These refinements were implemented for the third round of applications.

Maintaining regional balance has been a critical element of the ADDRF program. However, our experiences show that the vast majority of applicants have been from Anglophone Africa and in particular, from South Africa, Nigeria, Kenya, and Uganda. In the first call for applications, we received a relatively large number of applications from Cameroon (15), which though bilingual (Anglo- and Francophone), has primarily had Francophone applicants. However, none of the applications from Francophone Africa were successful in the first round. Reflecting back, several possible reasons may have accounted for the low success of French-speaking applicants: First, the fellowship announcement was in English, which may have reduced coverage and reach in Francophone Africa. Second, although applicants could send applications in French, we did not state this clearly in the application form. Consequently, majority of French-speaking candidates submitted English translations that were often poorly prepared and written, which invariably reduced their chances of selection. Having noted this, the calls for second and third rounds of applications were issued in both English and French and applications were invited in both languages. While this has led to increased staffing demands because a bilingual person has to assist with the correspondence and translation of documents, it has resulted in a large increase in the number of French-speaking applicants (a total of 30 applications from 7 countries in the second round; and 31 applications from 10 countries in the third) as well as in the selection of five of these applicants for funding in the second round and two in the third. Our efforts to effectively reach Francophone African scholars, however, have also raised the need to ensure that the training workshops cater appropriately for a linguistically-diverse audience. Due to financial constraints, we cannot hold separate workshops for French and English speakers; thus, despite our best efforts to increase fellowship access to Francophone African and our having bilingual facilitators in the training workshops, those who benefit from the full program of activities are those who have a good working knowledge of English. Expansion to Lusophone African countries is also a high priority for the ADDRF program. However, budgetary, as well as staffing (in terms of bilingual readers) currently prevent us from accepting applications written in Portuguese.

A strong institutional base that fosters scholarship and research is critical for training and retaining the next generation of researchers and academics. Our experience suggests that efforts to build research capacity in African universities need to also specifically target graduate-level research supervisors, perhaps through mentorship or faculty development initiatives. Judging by the quality of many of the proposals we received, there is an apparent lack of strong supervision among majority of doctoral candidates, demonstrated, for instance, in significant flaws in the methods section of many proposals, poor writing skills, reliance on dated references, and in the poor conceptual capacity of the students. Moving forward with the ADDRF, we are keen to establish a mechanism to better assess the research environment (including departmental commitment to facilitate timely completion of the dissertation). One suggestion is to have the heads of departments provide details on available infrastructure and resources (e.g. internet access, staffing) at the university. However, the bureaucratic processes at many universities are likely to hinder timely provision of this information. In the third call, we required all students to submit their primary supervisor's curriculum vitae. Further, we are exploring the possibility of inviting Fellows' supervisors to the training workshops for sessions on effective supervision of graduate students.

Sound financial management is key to the program's sustainability. As such, we must ensure close follow-up of fund use by Fellows. Further, inculcating budgeting and accounting skills is an integral part of training students in research management thus, we require rigorous reporting and accounting for funds disbursed to students. While receiving the first progress and financial reports from the first cohort (which were required six months after the initial disbursement of funds), we realized that many were unfamiliar with reporting requirements. Progress reports ranged from scanty to overly detailed narratives while financial reports lacked the necessary support upon which further disbursement was contingent. This led us to develop templates for both progress and financial reports that would allow Fellows to include all the necessary details while simplifying the process for them. The progress report template, therefore, captures details on the progress of Fellows' work, timeline to completion, challenges faced (if any), any conferences attended or papers published, and expected date of graduation. This also allows us monitor more closely the progress of the Fellows, not just in their current research, but also in the development of their career as researchers. The financial reporting template has simplified the financial reporting for the Fellows and made it easier for us, administratively, to expedite the disbursement process. Fellows also receive training on reporting during their first workshop.

The fellowship program aims to address existing knowledge gaps in health systems research in sub-Saharan Africa. Ideally, funded studies should demonstrate clear linkages to relevant national policies and strategies and show great promise of making substantive contribution to strengthening health systems in Africa or should address cutting-edge issues in the field of sexuality (for Ford Foundation-funded applications). The fellowship calls specify that dissertation research may address any of the following issues: health sector analysis; health management and organization; disease burden; health care financing mechanisms (including health insurance); quality of care; human resources for health; program evaluation; health equity; research to practice; information, education and communication; health policies processes (including decentralization); and health information systems, among others. While the need and demand for fellowships targeting health issues, broadly defined, is great, there is good reason to narrow the scope of the program specifically to research on health systems. This is particularly important since one of our ultimate goals is to facilitate substantive contributions, by African scholars, to strengthening health systems in Africa.

As a new program, we actively request feedback on processes and program activities. For example, during the application review committee meeting, all reviewers have the opportunity to provide feedback on ways to improve the review process. Some of the changes to the application forms, for example, have been based on this feedback. In addition, following each training workshop, Fellows are requested to complete an evaluation form rating different aspects of the workshop (workshop content and design; utility of the topical area; and suggestions for improving the course). For example, in the scientific writing workshop organized for the first cohort of students, participants were asked to rate the usefulness of each workshop session on a 4-point Likert-type scale ranging from *not at all useful *to *very useful*. Thirty-five percent (n = 6) and 65% (n = 11) rated the overall workshop as useful and very useful, respectively. Our own self-evaluation has also proved helpful. For example, during the initial training workshop, we noted that many students were not only unfamiliar with reference management software, but the cost of such software was also prohibitive. Subsequently, we approached one of the software developers and negotiated a discounted bulk purchase for Fellows. In addition, we included a training session on the software.

## Conclusions

Underperformance of many tertiary institutions in Africa following economic and political crisis from the 1970's to 1990's led to widespread withdrawal of funding for tertiary education by many international agencies and funders. Further, structural adjustment programs forced many African governments to reduce investments in tertiary education [6]. However, increasingly, attention is being focused on the importance of research capacity for development. Unfortunately, many post-graduate programs are still under-funded. Thus, it is of little surprise that there is enormous demand for financial support among doctoral candidates at African universities. For instance, our initial call attracted 118 applications from over 15 countries in East, West, South, and Central Africa. Less than a fifth of these were funded. Building on the successful launch of the ADDRF, we successfully sought funding to support the program for the next two years (2009-10). The second call attracted 162 applications from 19 countries while the third attracted 134 applications from 23 countries. The large number of applications attracted by this relatively new program persuades us that taking the ADDRF and other fellowship programs rapidly to scale is essential to fostering long-term health research capacity in Africa, developing and retaining a critical core of highly-skilled researchers in the health field who are able to engage in active research, and re-establishing the African university as a key player in the region's development agenda. Underscoring the importance of funding, one fellow, who has recently graduated, stated:

"The ADDRF came at a critical point in my life as a doctoral student. I did not know how or who was going to help me meet most of the costs during my last two years of study as I was sponsoring my own studies. I tried to get part-time work to assist me pay for the required fees, accommodation costs, and most importantly, research as well as most of the costs needed for a propitious environment for academic engagement. But when the APHRC offered me the fellowship, it was like manna from heaven. And within the stipulated time, I was able to concentrate on my studies and beat the deadline by a good margin." (2008 Fellow, male)

Overall, fellows, reviewers, and program staff have indicated great satisfaction with this program. In one recipient's own words:

*"When I was selected to be part of the first set of ADDRF fellows, little did I know that it was the beginning of great things for my career. Through the fellowship grant, I was able purchase a laptop which was quite useful for my thesis writing... attended two workshops... the Population Association of America (PAA) conference in USA and the International Conference on Urban Health (ICUH) in Kenya where I presented some of the findings from my study. Attending these conferences gave me the opportunity of meeting and interacting with some scholars whose work I have read in learned journals." *(2008 Fellow, female)

The most critical outcome of the program will be its long-term impact in enhancing high-quality research outputs. With the monies currently available to support the program, the fellowship is expected to support 65 doctoral dissertations from African universities by 2012. Since Fellows are expected to publish at least one peer-reviewed article from their research within 36 months of receiving the award, we anticipate significant contributions to the body of knowledge in health, population, and sexuality research. Currently, five Fellows out of the 20 funded in the first round and two Fellows from the 25 funded in the second round have successfully defended their dissertations. Altogether, the first cohort of Fellows have given over 40 oral and poster presentations at regional and international conferences, and have submitted or published at least 15 papers in peer-reviewed journals since 2008. Further evidence of the long-term impact will include the proportion of dissertations completed within 24 months of receiving the awards, the number of manuscripts from the dissertations that are published in peer-reviewed journals and the first destination of grantees upon graduation (i.e. field and geographic location of their job placement). These indicators will be monitored to determine the ultimate value of the fellowship program. For us, then, future challenges include how to attribute outcomes to the fellowship program, maintaining long-term contact with Fellows after they graduate, and ensuring that they remain active in research and teaching.

## Competing interests

The authors declare that they have no competing interests.

## Authors' contributions

All authors are involved in coordinating the ADDRF program. CWK, COI, and ACE contributed to the grant proposals submitted to IDRC and the Ford Foundation for funding to support the ADDRF program. CWK prepared the journal manuscript. COI, SWW, and ACE reviewed and edited the manuscript. All authors read and approved the final manuscript.
